# Thrombocytopenia and clinical outcomes among patients with COVID‐19 disease: A cohort study

**DOI:** 10.1002/hsr2.1111

**Published:** 2023-02-13

**Authors:** Hussain Alkhalifa, Zaenb Alsalman, Aman Alfaraj, Mohammed Algaraash, Mortadah Alsalman

**Affiliations:** ^1^ Department of Medicine, College of Medicine King Faisal University Saudi Arabia; ^2^ Department of Family and Community Medicine, College of Medicine King Faisal University Saudi Arabia; ^3^ Department of Medicine King Fahad Specialist Hospital Dammam Saudi Arabia; ^4^ Department of Medicine Prince Saud Bin Jalawy Hospital Alahsa Saudi Arabia

**Keywords:** acute kidney injury, COVID‐19 disease, mechanical ventilation, thrombocytopenia

## Abstract

**Background and Aims:**

Thrombocytopenia is increasingly recognized among patients with critical illness and plays a role in several diseases affecting different organ systems. Therefore, we studied the prevalence of thrombocytopenia among hospitalized COVID‐19 patients and its correlation with disease severity and clinical outcomes.

**Methods:**

This was an observational retrospective cohort study conducted on 256 hospitalized COVID‐19 patients. Thrombocytopenia is defined as a platelet count below 150,000/μL. Disease severity was classified based on the five‐point CXR scoring tool.

**Results:**

Thrombocytopenia was found in 66 (25.78%) patients. In outcomes, 41 (16%) patients were admitted to intensive care unit, 51 (19.9%) died, and 50 (19.5%) had acute kidney injury (AKI). Of the total patients with thrombocytopenia, 58 (87.9%) had early thrombocytopenia, while 8 (12.1%) had late thrombocytopenia. Notably, mean survival time was markedly decreased in late‐onset thrombocytopenia cases (*p* < 0.0001). Patients with thrombocytopenia showed a significant increase in creatinine compared to those with normal platelet counts (*p* < 0.05). Moreover, thrombocytopenia was more prevalent in patients with chronic kidney disease compared to other comorbidities (*p* < 0.05). In addition, hemoglobin was significantly lower in the thrombocytopenia group (*p* < 0.05).

**Conclusion:**

Thrombocytopenia is a common finding among COVID‐19 patients, with a predilection toward a specific group of patients, though the exact reasons are unclear. It predicts poor clinical outcomes and is closely linked to mortality, AKI, and the need for mechanical ventilation. These findings suggest that more research is required to study the mechanism of thrombocytopenia and the possibility of thrombotic microangiopathy in COVID‐19 patients.

## 1 INTRODUCTION

In late 2019, a new coronavirus appeared, leading to the greatest pandemic in recent decades. It has affected almost every country, causing a highly significant number of deaths. The disease was named coronavirus disease 2019 (COVID‐19); the causative virus is severe acute respiratory syndrome coronavirus 2 (SARS‐CoV‐2).^1^

Infections range from mild to severe or even lethal, with an overall case fatality rate of 2.3%.^2^ Diagnosis is based on a broad spectrum of clinical presentation, confirmed by direct detection of SARS‐CoV‐2 RNA by nucleic acid amplification tests. Nasopharyngeal and oropharyngeal swab tests are the standards for diagnostic specimen collection.^3^

Additional hematological laboratory findings include neutrophilia; lymphopenia thrombocytopenia is commonly found in hospitalized COVID‐19 patients.^4^, ^5^ These findings can be a valuable tool for predicting severity and outcomes for individuals affected by this condition.^6^, ^7^ Several studies have analyzed these abnormalities among COVID‐19 patients and correlated them with disease progression and mortality.^7^

Thrombocytopenia is defined as a platelet count below the lower limit of normal (150,000/μL), and it is a common hematological laboratory finding in COVID‐19 patients, with a prevalence between 5% and 41%. Several mechanisms have been indicated in how COVID‐19 may result in thrombocytopenia. One possibility is that COVID‐19 either directly attacks the hematopoietic stem cells or induces an autoimmune phenomenon. Another possibility is that thrombocytopenia occurs in COVID‐19 patients due to an irregular bone marrow microenvironment and decreased thrombopoietin production, an abnormal local renin‐angiotensin system, and other inflammatory cytokines. A third potential explanation is that COVID‐19 lung damage leads to thrombocytopenia since the lungs play a crucial role in platelet biogenesis and act as a reservoir for immature megakaryocytes.^5^, ^8^

In the current study, we examine the prevalence of thrombocytopenia among hospitalized COVID‐19 patients and its correlation with disease severity, complications, and hospital mortality.

## 2 MATERIALS AND METHODS

### 2.1 Study design and participants

This is an observational retrospective cohort study, which enrolled hospitalized confirmed COVID‐19 patients from Prince Saud Bin Jalawy Hospital (Al‐Ahsa, Saudi Arabia) to assess the prevalence of thrombocytopenia and its relationships to illness severity and clinical outcomes. Medical records were used to collect patients’ demographic, basic clinical and laboratory data, type of respiratory support, and outcomes. Patients who did not have access to the electronic medical record were under the age of 16, had immune thrombocytopenic purpura, or had been directly admitted to the intensive care unit (ICU) were all excluded. This study was approved by King Fahad Hospital's Ethical Committee (KFHH RCA 35‐42‐2021). In addition, consent from Prince Saud Bin Jalawy Hospital was obtained to utilize the anonymous data for the study.

### 2.2 Definitions

Participants were labeled as having delayed thrombocytopenia if they developed thrombocytopenia 30 days after discharge or experienced thrombocytopenia in a second admission postdischarge.

Disease severity was classified based on the extent of lung involvement shown on chest X‐ray, using a validated chest X‐ray score from 1 to 5: 1 is average; 2 shows patchy atelectasis, and/or hyperinflation, and/or bronchial wall thickening; 3 shows focal alveolar consolidation involving no more than one segment or one lobe; 4 shows multifocal consolidation; and 5 shows diffuse alveolar consolidation.^9^

The clinical outcome was assessed based on the patient's discharge, ICU admission, mechanical ventilation (MV) requirement, development of organ failure, thrombosis evidence on Doppler or CT or MRI imaging, and mortality.

### 2.3 Statistical analysis

Data entry, processing, and statistical analysis were carried out using MedCalc Ver. 20 (MedCalc). For descriptive statistics, mean and SD were used for continuous variables, while frequencies and percentages were utilized for categorical variables. Student's *t*‐test was used as a significance test for continuous variables, and the Chi‐square test was used for categorical variables. The receiver operating characteristic (ROC) curve analysis was used to evaluate the predictive value of thrombocytopenia for COVID‐19 outcomes. *p* Values less than 0.05 (5%) were considered statistically insignificant.

## 3 RESULTS

A total of 256 hospitalized COVID‐19 patients were retrospectively evaluated, with a median age of 55.8 ± 16.5, average oxygen (O_2_) saturation of 87 ± 10, and a mean platelet count of 258.12 ± 115.12 × 10^3^/µL. Out of these patients, 177 (68.7%) required noninvasive ventilation and 44 (17.2%) required MV, and 33 (12.9%) patients had no respiratory support. Other participants' baseline characteristics are shown in Table 1.

**Table 1:** Basic clinical data among 256 COVID‐19 patients.

Variables	Frequency (%) mean ± SD
Age (years)	55.8 ± 16.5
Gender	
Female	109 (42.6%)
Male	147 (57.4%)
Type of respiratory support	
None	33 (12.9%)
FM	71 (27.7%)
HFNC	13 (5.1%)
MV	47 (18.4%)
NC	44 (17.2%)
NRFM	48 (18.8%)
Comorbidities	
DM	127 (49.6%)
HTN	130 (50.8%)
CVD	32 (12.5%)
Stroke	10 (3.9%)
CKD	12 (4.7%)
Asthma/COPD	15 (5.9%)
SCD	8 (3.1%)
Solid CA	3 (1.2%)
Hematology CA	2 (0.8%)
Liver disease	2 (0.8%)
Baseline laboratory data	
Hemoglobin (g/dL)	12.43 ± 2.39
Leukocyte count (10^3^/µL)	7.45 ± 4.71
Platelets (10^3^/µL)	258.12 ± 115.12
LDH (mg/dL)	379.69 ± 194.98
D‐dimer (mg/dL)	4.38 ± 11.82
Creatinine (μmol/L)	129.19 ± 275.06

Abbreviations: CA, cancer; CKD, chronic kidney disease; COPD, chronic obstructive pulmonary disease; CVD, cerebrovascular disease; DM, diabetes mellitus; FM, face mask; HFNC, high‐flow nasal cannula; HR, heart rate; HTN, hypertension; LDH, lactate dehydrogenase enzyme; MAP, mean arterial blood pressure; MRFM, nonrebreather mask function; MV, mechanical ventilator; NC, nasal cannula; RR, respiratory rate; SCD, sickle cell disease.

Radiological data show that the average severe acute respiratory infections chest X‐ray (SARI CXR) score was (3.84 ± 1.09), with 3 (1.2%) patients having radiological evidence of thrombosis.

Outcome data show that the average length of hospital stay (LOS) was (8.9 ± 5.8) days, with 41 (16%) patients having ICU admission, 51 (19.9%) having suffered mortality, and 50 (19.5%) having acute kidney injury (AKI). In addition, thrombocytopenia was found in 66 (25.78%) patients, with no significant difference between male and female participants (Chi‐square test *p* > 0.05). Patients taking chloroquine, linezolid, carbapenems, glycopeptides (vancomycin), vasopressors, and fluoroquinolones were more likely to have thrombocytopenia (Chi‐square test *p* < 0.05).

Of the total patients with thrombocytopenia, 58 (87.9%) had early thrombocytopenia, and 8 (12.1%) had late thrombocytopenia. Notably, mean survival time was markedly decreased in late‐onset thrombocytopenia cases over an average of 20 days (log‐rank test *p* < 0.0001). However, there was a nonsignificant correlation with CXR score (Student's *t*‐test *p* > 0.05).

Patients with thrombocytopenia showed a significant increase in creatinine and blood urea nitrogen compared to those with normal platelet counts (Student's *t*‐test *p* < 0.05). Moreover, thrombocytopenia was more prevalent in those with chronic kidney disease (CKD) compared to other comorbidities (Chi‐square test *p* < 0.05). Anemia was found in 125 (48.82%) participants. In addition, hemoglobin was significantly lower in the thrombocytopenia group (Student's *t*‐test *p* < 0.05). Lactate dehydrogenase enzyme (LDH) levels and nadir PT, nadir PTT as well as INR were higher in patients with thrombocytopenia, but this was not statistically significant (Student's *t*‐test *p* > 0.05). In addition, the mortality rate and need for MV were significantly higher in patients with thrombocytopenia (Chi‐square test *p* < 0.05). However, LOS, thrombosis outcome rate and radiology evidence of thrombosis were similar between both groups (Chi‐square test *p* > 0.05).

**Figure 1:**
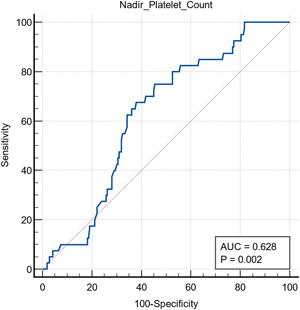
Receiver operating characteristic curve analysis showing thrombocytopenia as a predictor for intensive care unit admission area under the curve (AUC) = 0.628.

**Figure 2:**
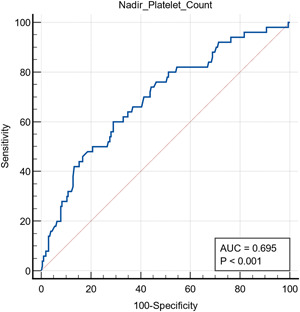
Receiver operating characteristic curve analysis showing thrombocytopenia as a predictor for mortality. Area under the curve (AUC) = 0.695.

**Figure 3:**
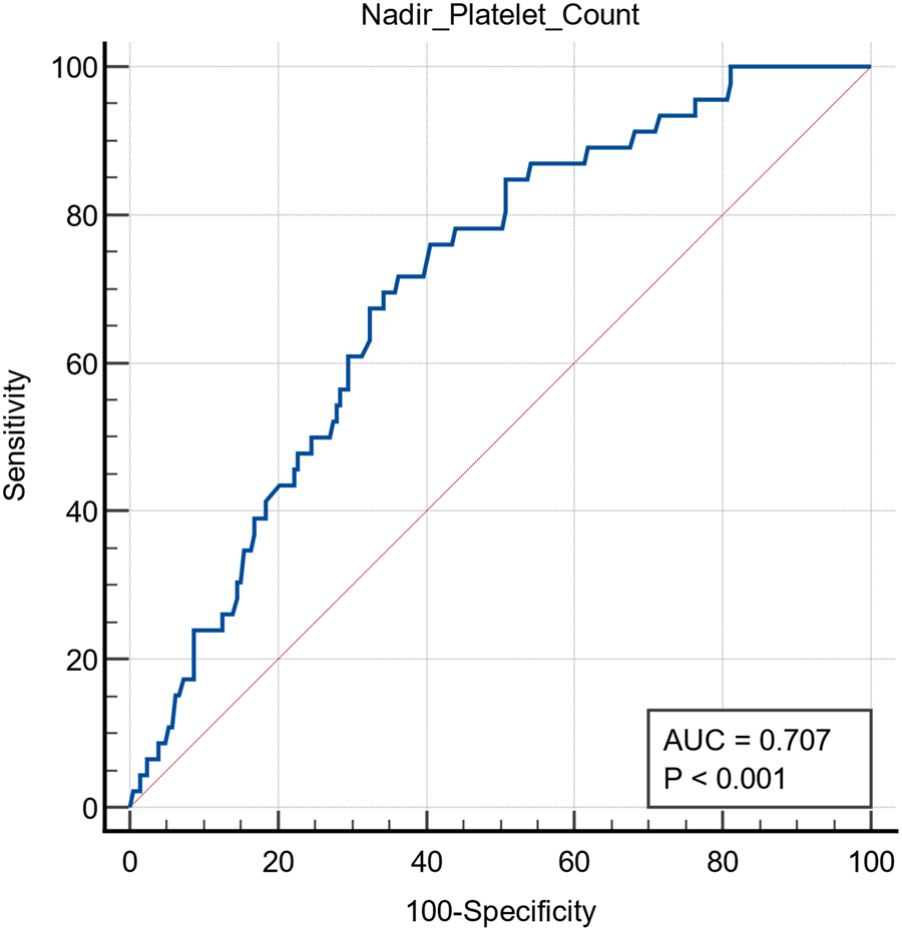
Receiver operating characteristic curve analysis showing thrombocytopenia as a predictor for need for mechanical ventilation. Area under the curve (AUC) = 0.707.

**Figure 4:**
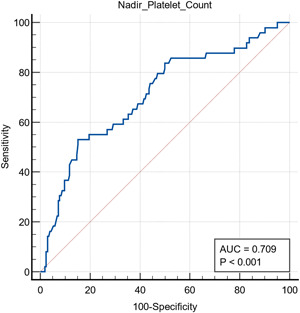
Receiver operating characteristic curve analysis showing thrombocytopenia as a predictor for acute kidney injury. Area under the curve (AUC) = 0.709.

ROC curve analysis was conducted, and thrombocytopenia showed nonsignificant predictive values for thrombosis (*p* > 0.05). However, it did predict ICU admission, mortality, MV, and AKI, though accuracy is variable (*p* < 0.05; 62%, 85%, 83%, and 85%, respectively; see Figures 1, 2, 3, 4).

## 4 DISCUSSION

A platelet is a small molecule that has multiple, diverse interactions with its surrounding environment. While the role of platelets in hemostasis and thrombosis is pivotal, they also have important functions related to immune responses, angiogenesis, atherosclerosis, and inflammation.^10^, ^11^ Furthermore, they play a role in several diseases affecting different organs and organ systems. Thrombocytopenia is increasingly recognized among patients with critical illness, and various studies found it in 35%–45% of patients in the ICU.^12^, ^13^

Previous reports revealed that COVID‐19 patients with thrombocytopenia likely require ICU admission and MV support. Furthermore, they exhibit worse outcomes and an increased risk of death.^14^, ^15^ In our study, we found that the prevalence of thrombocytopenia in hospitalized COVID‐19 patients was around 25%, which is slightly higher than previously reported among patients affected with COVID‐19 and a thousandfold that caused by COVID‐19 vaccines.^16^, ^17^ 12% of our participants developed late thrombocytopenia. Thrombocytopenia was shown to be a significant predictor of ICU admission, need for MV, and mortality. Interestingly, patients with late‐onset thrombocytopenia, particularly those over an average of 20 days, show a markedly decreased survival rate, which is consistent with previous reports.^14^

AKI is not uncommon among COVID‐19 patients; it is found in 5.1% of affected individuals during hospitalization, though the mechanism is unclear. Furthermore, patients with underlying CKD and 30‐day survivors of COVID‐19 exhibited a higher risk for AKI.^18^, ^19^ In this work, we found that around 20% of participants developed AKI, which is around fourfold the amount previously reported.^19^, ^20^

Interestingly, thrombocytopenia was closely linked with and significantly predicted AKI, in contrast to previous reports.^21^ Moreover, patients with CKD were more likely to develop thrombocytopenia.

Hematological abnormalities are common features of COVID‐19. Anemia was identified in 61% of COVID‐19 patients.^6^ In our study, we found that patients with thrombocytopenia had lower hemoglobin levels compared to those with normal platelet counts. In addition, these patients were more likely to have high LDH, but this did not reach statistical significance. Moreover, the presence of thrombocytopenia with increased D‐dimer and prolonged coagulation time (PT and PTT) increased suspicion of disseminated intravascular coagulation (DIC). However, many studies report that most COVID‐19 patients do not meet the typical DIC criteria.^22^, ^23^

Implications of our study findings emphasize that thrombocytopenia is multifactorial and associated with poor clinical outcomes. Nevertheless, constellations of these findings, including thrombocytopenia in the context of AKI and anemia, raise the possibility of localized as well as thrombotic microangiopathy, which was previously suggested based on anecdotal reports.^20^, ^23^ However, this theory is limited and not fully proven, due to a lack of peripheral blood smears and tissue biopsies.

In conclusion, thrombocytopenia is a frequent finding in COVID‐19 patients, with a predilection towards certain groups of patients, though the exact reasons are unclear. It predicts poor clinical outcomes and is closely linked to mortality and the need for MV. Further studies are needed to investigate the underlying mechanism of thrombocytopenia in COVID‐19 and whether certain measures improve it and ameliorate the clinical course of the disease through early recognition and interventions.

## AUTHOR CONTRIBUTIONS

**Hussain Alkhalifa**: Conceptualization; data curation; writing—original draft; writing—review and editing. **Zaenb Alsalman**: Data curation; formal analysis; writing—review and editing. **Aman Alfaraj**: Conceptualization; data curation; formal analysis; writing—review and editing. **Mohammed Algaraash**: Data curation; formal analysis; writing—review and editing. **Mortadah Alsalman**: Conceptualization; data curation; formal analysis; writing— original draft; writing—review and editing.

## CONFLICT OF INTEREST STATEMENT

The authors declare no conflict of interest.

## TRANSPARENCY STATEMENT

The lead author Zaenb Alsalman affirms that this manuscript is an honest, accurate, and transparent account of the study being reported; that no important aspects of the study have been omitted; and that any discrepancies from the study as planned (and, if relevant, registered) have been explained.

## Data Availability

The data used and analyzed during this study are available from the authors on reasonable request.
